# In Situ Characterization of the Reaction-Diffusion Behavior during the Gradient Interphase Formation of Polyetherimide with a High-Temperature Epoxy System

**DOI:** 10.3390/polym14030435

**Published:** 2022-01-21

**Authors:** Lucian Zweifel, Christian Brauner, Julie Teuwen, Clemens Dransfeld

**Affiliations:** 1 Institute of Polymer Engineering, FHNW University of Applied Sciences and Arts Northwestern Switzerland, Klosterzelgstrasse 2, 5210 Windisch, Switzerland; christian.brauner@fhnw.ch; 2 Aerospace Manufacturing Technologies, Faculty of Aerospace Engineering, Delft University of Technology, 2629 HS Delft Delft, The Netherlands; j.j.e.teuwen@tudelft.nl (J.T.); c.a.dransfeld@tudelft.nl (C.D.)

**Keywords:** thermoset resin, thermoplastic, interphase formation, characterization, optical properties/techniques, Raman spectroscopy, reaction-diffusion

## Abstract

This study presents two novel methods for in situ characterization of the reaction-diffusion process during the co-curing of a polyetherimide thermoplastic interlayer with an epoxy-amine thermoset. The first method was based on hot stage experiments using a computer vision point tracker algorithm to detect and trace diffusion fronts, and the second method used space- and time-resolved Raman spectroscopy. Both approaches provided essential information, e.g., type of transport phenomena and diffusion rate. They can also be combined and serve to elucidate phenomena occurring during diffusion up to phase separation of the gradient interphase between the epoxy system and the thermoplastic. Accordingly, it was possible to distinguish reaction-diffusion mechanisms, describe the diffusivity of the present system and evaluate the usability of the above-mentioned methods.

## 1. Introduction

In aerospace, fiber-reinforced composite materials are increasingly used due to their potential of significantly reducing weight and improving mechanical performance. Furthermore, fiber-reinforced composites help solve the environmental challenges of sustainable mobility [1,2,3]. Carbon fiber reinforced polymers (CFRPs) based on thermosetting epoxy matrix systems exhibit exceptional strength and stiffness at a low weight and therefore find increasing success in large aircraft structures. Commonly, such components are cured in an autoclave at high pressure and temperature to achieve high quality and reproducibility. Beyond their processing advantages, highly cross-linked thermosets tend to be brittle with low resistance to crack initiation and growth [4,5]. Therefore, several strategies have been thought of to improve the fracture toughness of epoxy systems [4,5,6,7]. The general concept proposes incorporating a second polymer in the epoxy matrix that is used as a toughening agent to prevent the crack propagation, thus leading to the improvement of the fracture toughness [5]. Several thermoplastics have been studied to toughen high-performance aerospace epoxy systems, such as poly(hydroxy ether) (phenoxy), poly(ether sulfone) (PES), poly(ether imide) (PEI) and poly(etherether ketone) (PEEK) [4,6,7,8,9]. The formation of interpenetrating polymer networks (IPN) and phase separating morphologies is an essential mechanism to improve fracture toughness [4,9,10].

The use of thermoplastics may also have benefits in joining thermoset composite structures. So far, joining of composite has mostly relied on traditional joining technologies, such as adhesive bonding or mechanical fasteners, which both have drawbacks. Joining with adhesives results in high costs and long processing times. Thermoplastic welding offers the ability of melting and reprocessing compared to thermosets, which cannot be re-melted after cross-linking. Additionally, thermoplastic welding allows fast processing speeds without significant surface preparation efforts, resulting in strong and dependable mechanical performance [11,12,13].

A novel approach to using the thermoplastic welding process for thermosets is to co-cure a thermoplastic interlayer during the cure process of a thermoset composite. Despite the fact that only the thermoset resin undergoes a curing reaction, that process is referred to as ‘co-curing’ in the literature [11,14,15,16,17,18,19]. For consistency with already published research, this term is also used in the present paper. This enables a functional surface that can be processed with thermoplastic welding processes such as resistance welding [13,20,21,22] and ultrasonic welding [11,14,23]. A gradient interphase forms between the epoxy resin and the thermoplastic, whereby the two components partially dissolve, diffuse and finally lead to a reaction-induced phase separation. It was shown in recent studies that polyetherimide (PEI) is a suitable candidate for interphase formation in aerospace-related applications [12,14,24,25,26]. The result is a pronounced heterogeneous morphology with a thermoplastic-rich phase and a thermoset-rich phase, including strong mechanical interlocking for subsequent load transfer [25]. The stages of the gradient interphase formation are further elaborated due to their significant influence on the final properties. During curing, the initially soluble materials are in contact at a temperature typically below the thermoplastic material’s glass transition. The initial solubility implies that mutual diffusion mechanisms occur [25]. First, the two components come into contact whereby the thermoplastic starts to swell and increases in volume due to penetration of the epoxy monomer molecules diffusing through it. During dissolution, the components of the thermoset resin disentangle and dissolve the thermoplastic polymer chains. Then, simultaneous diffusion of both materials into each other occurs. Here, Fickian diffusion kinetics, e.g., Fickian (Case I) [27], non-Fickian (two stage sorption, Case II and Super Case II) [27,28,29,30] and anomalous diffusion [28,29], could help to understand the apparent transport phenomena. It is noteworthy that Fick’s law cannot describe many polymers, especially when a low-molecular weight solvent causes extensive swelling of the polymer. This is the case with glassy polymers, which exhibit non-Fickian (Case II) behavior with a sharp front in the concentration profile that advances linearly in time [29]. Super Case II includes additional characteristics in comparison to Case II, such as an induction period at the beginning of the process [30]. Hence, it is necessary to take into account the time-dependent response of a polymer [29]. At the beginning of curing, high diffusion rates due to low molecular weight are apparent. Curing continues, the molecular weight increases and the ability to diffuse decreases. The ongoing curing induces a shifting of the critical solution temperature, which eventually leads to phase separation. Here, the thermodynamic stability of the constituents influences the miscibility gap. Depending on the material combinations, the miscibility gap can be characterized by an upper critical solution temperature (UCST) [10,25] or a lower critical solution temperature (LCST), which both indicate an area of partial miscibility or miscibility for certain compositions only [10]. Within this region, two separation mechanisms can occur, such as spinodal decomposition, leading to co-continuous morphologies or nucleation and growth, depending on concentration, time, temperature and pressure. The higher the temperature, the later the onset of reaction-induced phase separation due to higher chain mobility and solubility [24]. The interphase becomes larger as the diffusion rate prevails over the reaction rate of the thermoset. The interphase eventually reaches its maximum extent when the degree of cure of the thermoset system limits the diffusivity. This phenomenon is further referred to as reaction-diffusion behavior. On the contrary, if there are non-reactive components (e.g., solvent diffusing into a thermoplastic), it is further referred to as diffusion behavior.

So far, the following methodologies have been used for characterizing interphase formation, where most only provide qualitative information of the reaction-diffusion process: hot stage microscopy for identifying physico-chemical properties [12,24,25,31]; analysis of interphase morphology depending on epoxy and hardener concentration ratios by scanning electron microscopy [11,12,14,24,25,32]; energy-dispersive X-ray spectroscopy [33] and micro-Raman spectroscopy [34]; the measurement of the interphase thickness with Raman spectroscopy [20,24]; and mechanical assessment of welded samples by lap shear strength and fracture analysis [11,12,14,21,22,35]. Additionally, it is important to mention attenuated total reflection Fourier transform infrared spectroscopy (ATR FTIR). The benefits of this technique are that the diffusion, swelling and reaction can be measured in situ, and the diffusion of each component can be monitored independently by monitoring the specific absorption bands in the infrared region [36,37,38,39,40]. ATR FTIR and Raman spectroscopy are fast, non-destructive and real-time analytical methods for analysis of molecular structure properties and their changes in reactive environments [41] with high spatial resolutions. Both in situ methodologies present distinct advantages and drawbacks [41,42,43,44], e.g., glass is a weak Raman scatterer, allowing simple constructions of in situ Raman cells to study catalysts at higher temperatures [42,45]. In comparison to ATR FTIR, Raman spectroscopy offers simpler sample preparation combined with confocal imaging, which makes it an ideal methodology for real-time monitoring in this study. Hence, in situ Raman technology is an ideal measurement technique available to date, allowing a time- and spatially-resolved in situ characterization of the interphase formation during the co-curing process. Such methods would enable a better understanding to tailor desired gradient interphase with the aim to determine the formation and microstructure.

This work aims at assessing and linking methodologies for in situ characterization of the reaction-diffusion process during the co-curing of a PEI thermoplastic interlayer with an epoxy-amine thermoset that allow combining spatial and temporal resolution. First, a computer vision point tracker algorithm was developed to follow and quantify diffusion fronts during optical hot stage microscopy. Second, an in situ Raman methodology was introduced whereby the hot stage setup was coupled with a Raman spectrometer, which enables time- and temperature-dependent measurements at defined positions, whereby the spectral change over time leads to a detailed analysis of diffusion and reaction-diffusion within the interphase formation. Finally, the diffusion rates of hot stage microscopy and in situ Raman spectroscopy were compared to draw conclusions of their accuracy and usability.

## 2. Materials and Methods

### 2.1. Materials

A high-performance epoxy system with a glass transition temperature around 180 °C provided by Huntsman Advanced Materials (Basel, Switzerland) was used. It is based on bi- and trifunctional blends of triglycidized meta-aminophenol (TGMAP) and bisphenol F diglycidyl ether (DGEBF) as resin, and 3,3’-diaminodiphenylsulfone (DDS) as an amine hardener. The bisphenol F component is essential for lowering the viscosity of the epoxy system, with a viscosity of 1200–1800 mPas at 25 °C [46]. It is characteristic for base matrix resin, as is found in aerospace grade prepreg systems [47]. The mixing ratios were defined as follows: 49.8% TGMAP, 13.7% DGEBF and 36.5% DDS. Additionally, a cure kinetic for the epoxy system was derived (see Supplementary Materials, Table S1) to correlate physico-chemical events during the experiments. The blend of the epoxy monomers of TGMAP and DGEBF is further referred to as epoxy precursor. The DDS is referred to as amine precursor. Both of them are also referred to as (non-reactive) monocomponents. The reactive mix of TGMAP and DGEBF epoxy blend with the DDS amine hardener is further referred to as (reactive) multicomponents.

PEI Ultem 1000, by Sabic, an amorphous thermoplastic having a high glass transition temperature at 217 °C with a weight average molecular weight of 55,000 g/mol (*n* ≈ 90), was used as a thermoplastic film with a nominal thickness of 125 μm. The measured average thickness was 126.8 μm with a standard deviation of 0.94 μm (0.74%). The films were dried in a convection oven for 5 h at 150 °C before the experiments to reduce the humidity take-up during storage and preparation [48].

### 2.2. Optical Hot-Stage Microscopy Coupled with a Point Tracker Algorithm

**Figure 1:**
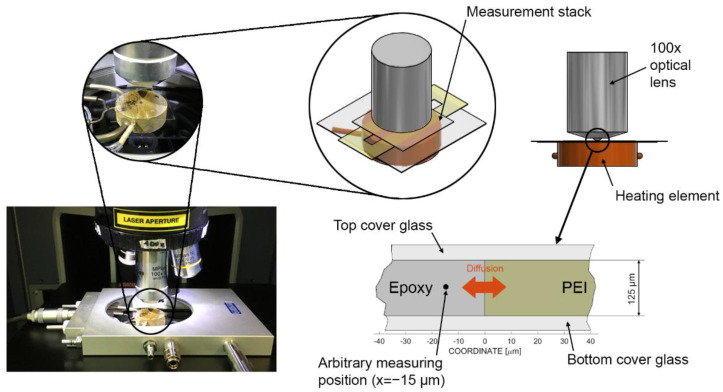
Adapted measurement stack of the in situ Raman spectroscopy coupled with hot stage microscopy including a thermoplastic film with an epoxy gap.

To measure the interphase formation and characterize its microstructural properties as a function of temperature, an optical hot-stage microscopy setup was used, as used by Teuwen and Farooq et al. [9,12,24], where a controlled heating device (Linkam THMS600, Tadworth, Great Britain) was coupled with optical microscopy (Keyence VHX 600, Osaka, Japan). Figure 1 shows the adapted measurement stack. The temperature was regulated by the heating element and was assumed to remain constant between the bottom and the top cover glass. The thermoplastic films with a nominal thickness of 125 μm were cut to 10 × 30 mm^2^ with a rectangular gap of 3 × 20 mm^2^. First, each thermoplastic film was heated at a rate of 50 K min^−1^ to 260 °C (above the glass transition temperature of 217 °C) while applying pressure to create intimate contact between the cover glasses and film. Then, the temperature was decreased to the curing temperature, which varied between 120 °C and 220 °C. As soon as the cure temperature was reached, a droplet of the epoxy components or the mixed epoxy system was dripped into the gap in the thermoplastic film. The epoxy would spontaneously fill the cavity by capillary action. First, monocomponent diffusion experiments were performed with the optical hot stage microscopy set-up. Measurements were executed for epoxy and amine precursors at different temperatures (120 °C to 220 °C). Second, multicomponent diffusion experiments were performed based on the same specifications. This setup is further referred to as ‘reactive multicomponent’, referring to multiple diffusional and reactional effects that arise simultaneously. A time-lapse program was used to capture an image every 15 s to characterize the interphase and to quantify the diffusion length at different temperatures of the PEI and epoxy system.

**Figure 2:**
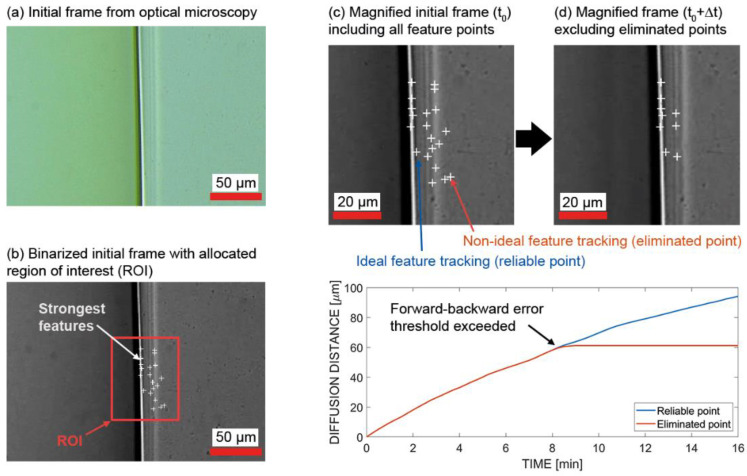
Identification of robust features and extraction of reliable tracking points: (**a**) initial frame at the beginning of diffusion from optical microscopy without post-processing; (**b**) binarized initial frame with allocated region of interest (ROI) in MATLAB; (**c**) magnified initial frame at diffusion step t_0_ including strongest feature points detected by Shi–Tomasi corner detector algorithm; and (**d**) magnified frame at diffusion stept_0_ + ∆t, excluding eliminated points due to exceedance of the forward–backward error threshold.

The Kanade–Lucas–Tomasi (KLT) algorithm implemented in MATLAB Computer Vision Toolbox was used to select features and align (track) image patches in a defined area of the diffusion front [49]. The algorithm is based on the Shi–Tomasi corner detector algorithm [50], which directly computes the eigenvalue decomposition under the assumption that corners are more stable for tracking. This method is also referred to as the Kanade–Tomasi corner detector [51,52,53]. The point tracker works particularly well for tracking objects that do not change shape over time, which proved satisfactory for the diffusion experiments. The algorithm consisted of the following steps: First, the initial frame with the size of 1600 × 1200 pixels (72 dpi) was imported, and the region of interest was specified. Then, the twenty strongest features were identified and extracted based on the eigenvalue interpretation of the Shi–Tomasi corner detector algorithm [50]. Then, the points were tracked, respectively aligned within every microscopy image provided as an input. Here, the coordinates in x and y directions of each frame were added in a control variable. The relative change in position in the y direction represents the diffusion front. Additionally, a forward-backward error threshold of 5 pixels (equal to 1.89 μm) was utilized to effectively eliminate points that were not tracked reliably. As the point tracker algorithm progressed over time, points on the diffusion front were lost at times due to brightness variations. Therefore, images were binarized to black and white, and brightness was normalized, ensuring that the points were identifiable through the whole experiment by the point tracker algorithm. Many points did not show an adequate representation of the diffusion line. Thus, curves were only extracted if the tracking coordinate increased continuously until the end of the diffusion process, meaning the forward-backward error threshold was not exceeded (see Figure 2). With this approach, one representative curve for each temperature was collected. Figure 2 shows the initial frame, conversion and allocation of the region of interest followed by the distinction between robust points and eliminated points.

### 2.3. Raman Spectroscopy Coupled with In Situ Hot Stage Microscopy

To perform in situ diffusion measurements (see Figure 1) as a function of time and temperature, the optical hot stage microscopy set-up was coupled with a confocal Raman microspectrometer (Horiba XploRA™ PLUS, Kyoto, Japan). Its spatial resolution in the x and y direction was <500 nm and in z < 2 μm, and its spectral resolution (full width at half maximum) was 1.4–8 cm^−1^, depending on the laser and grid. The 100x lens with a minimum working distance of 0.21 mm was used, which was essential when dealing with multi-layered samples. The defined target was to map Raman spectra at a specific position over the period of 3000 s, where a spectrum was recorded every 30 s. This enabled the measurement of relative concentrations that are temperature and time dependent. The relative intensity of Raman peaks is directly proportional to the relative concentration of the components in a sample [54]. Each experiment was repeated four times to ensure statistical validity. The experimental procedure was as follows: After preparing the sample as in the previous section, a suitable interface position was chosen with the 100× lens. Here, the x, y and z coordinates at the thermoplastic interface edge were set to zero, and an x-offset with respect to positive and negative direction was applied. Then, the chosen position was exposed to the laser for 2 min (photobleaching method [55]). Next, the video function was activated to confirm that the film did not move, and the epoxy cavity was visible. Finally, the cavity was filled with an epoxy precursor or a mixed epoxy resin droplet, and Raman time mapping with a spectra every 30 s for 50 min was started. Table 1 presents an overview of the parameters used for the time-mapped experiments.

**Figure 3:**
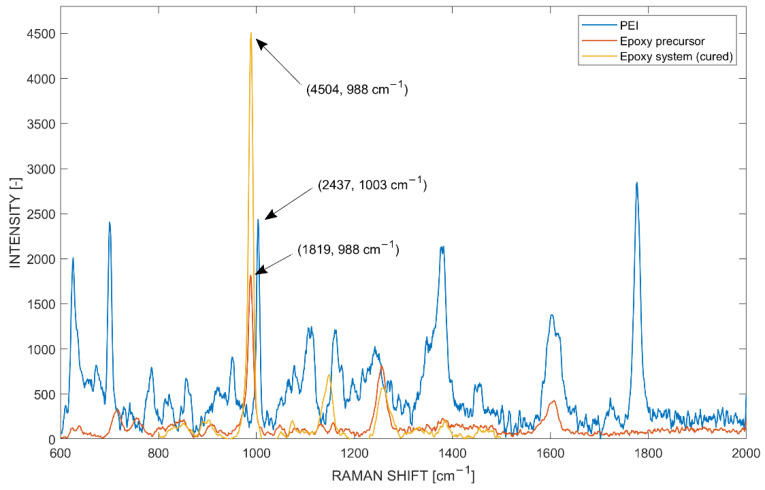
Reference spectra for normalization of PEI, epoxy precursor and epoxy system signals.

Data analysis of the measured spectra was performed using LabSpec6 software (Version 6.5, Horiba, Kyoto, Japan). Hereby, two characteristic peaks within each spectrum were identified by calculating a maximum peak value within a defined Raman shift range: for PEI between 998 and 1013 cm^−1^, and for the epoxy precursor and epoxy system between 980.6 and 995.6 cm^−1^ (Figure 3). The peak was identified by adjusting a two-point baseline to the curve and evaluating the peak value with respect to the baseline. Next, the data were normalized to a reference value of the peak intensity within a pure reference measurement. This allowed for the peak representation of pure reference measurements, the maximal achievable concentration being equal to 1. Other methods such as deconvolution of the spectra and classical least square fitting were evaluated but did not show any advantage in accuracy and consistency.

Characteristic band intensity peaks in the Raman spectrum were used to evaluate the relative change in intensity with respective to concentration over time. The mapping was used to investigate diffusion from PEI into the epoxy and vice versa. Reference spectra of pure PEI, pure epoxy precursor and the pure mixed epoxy system were measured in an identical measurement setup according to parameters in Table 1 (see Figure 3). Characteristic peaks of pure constituents or systems were used for the normalization of the mapped experiments; for PEI an intensity of 2437 (±0.7%) at 1003.45 cm^−1^, for epoxy precursor an intensity of 1819 (±0.75%) at 988.4 cm^−1^ and for the mixed epoxy system an intensity of 4504 (±0.68%) at 988.2 cm^−1^ were measured. The band peaks showed robust and repeatable intensity values. Further, the peaks fulfilled the requirement of having a band intensity 2 or 3 times greater than the intensity of the noise (limit of detection) [54].

## 3. Results

### 3.1. Mono and Multicomponent Diffusion Experiments with Optical Hot Stage Microscopy

**Figure 4:**
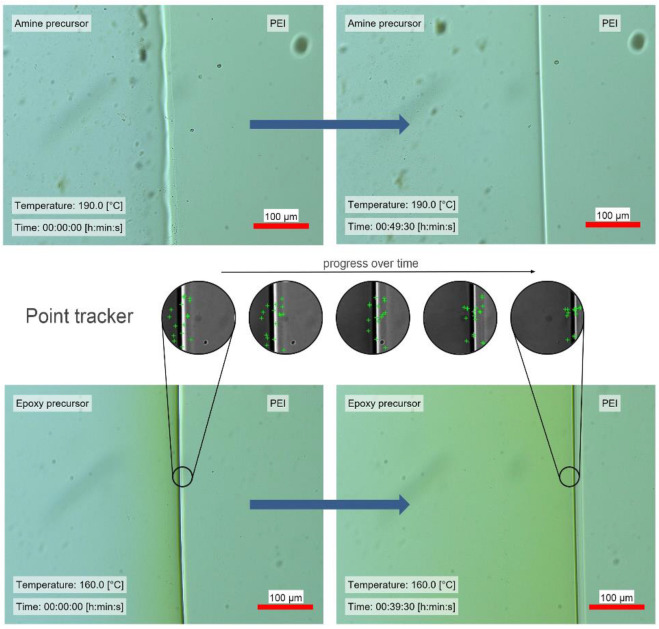
Monocomponent diffusion experiments of chosen precursors with PEI. From top to bottom: diffusion of amine precursor at 190 °C; point tracker image details and epoxy precursors at 160 °C, where the left image shows the starting time, and the right shows the diffusion after a defined time; diffusion of epoxy precursors at 160 °C.

**Figure 5:**
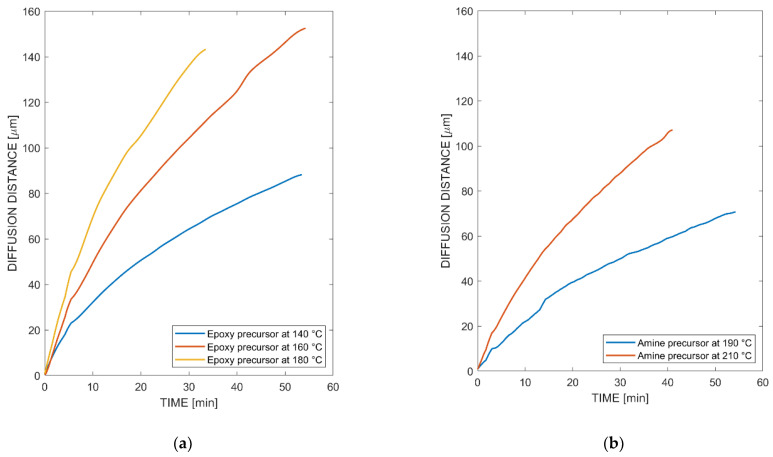
Diffusivity of monocomponent diffusion experiment on optical hot stage setup derived by point tracker algorithm: (**a**) epoxy precursor at various temperatures; (**b**) amine precursor at various temperatures.

Monocomponent diffusion experiments showed that the amine precursor, which initially was in a micro-pulverized form, started to melt between 160 and 180 °C. Hence, there was no dissolution below the temperature of 160 °C with PEI. For this reason, measurements were taken from 190 °C up to 210 °C to have a representative temperature range. The epoxy precursor allowed measurements starting from 120 °C. Figure 4 presents monocomponent diffusion experiments in which points were tracked to follow the diffusion front moving from left to right, hence tracking the progress of the amine precursor or epoxy precursor into PEI. Here, simultaneous diffusion, meaning diffusion of the monomers into the PEI, as well as the PEI diffusing into the monomers, was observed for both epoxy and amine precursors. Nevertheless, only one diffusion front from the epoxy precursor into PEI was tracked. The other diffusion front in the opposite direction was not clearly visible, and thus no robust visual features were extracted to make use of the point tracker. This was evidence of different diffusion mechanisms. Figure 5 presents the results where the diffusion length was plotted against the time measured. Again, the diffusion was only measured from the epoxy or amine precursor into PEI, which means that no information from the opposite side was available. Approximately 20% of the initially detected points with a robust feature were eliminated throughout the experiment. However, the succeeded points presented consistent results with an error equal to or lower than 1.89 μm (forward–backward error threshold). Generally, the diffusivity depends on temperature, free volume of the thermoplastic material, size and distribution of macromolecules and chemical interaction between the constituents [39,56,57]. Even though the molecular length and weight of TGMAP and DGEBF are higher in comparison to DDS, Figure 5 shows that the epoxy precursor displayed a higher diffusivity in comparison to the amine precursor. This is explained by a stronger chemical interaction between the epoxy precursor and PEI, as other influences such as free volume and temperature remained constant.

**Figure 6:**
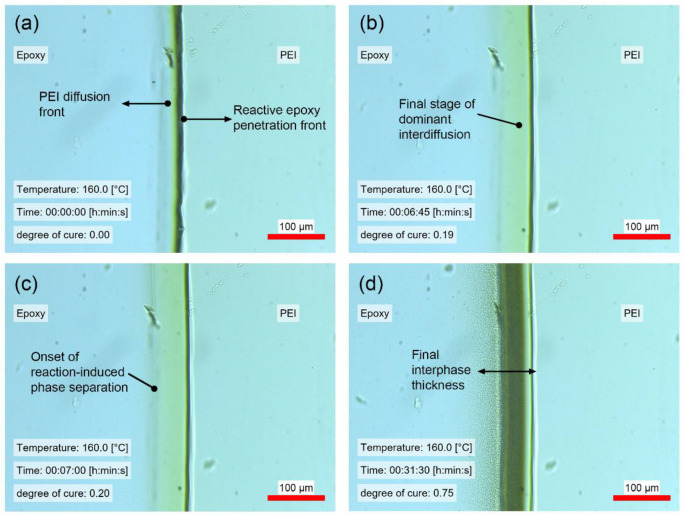
Reactive multicomponent diffusion experiment with the epoxy system at 160 °C: (**a**) start of partial dissolution; (**b**) end of diffusion; (**c**) initiation of reaction-induced phase separation; (**d**) end of phase separation with visible decomposed microstructure.

Next, the optical hot stage microscopy setup was used for reactive multicomponent diffusion experiments. Here, the point tracker algorithm was used to track the diffusion front until the reaction-induced phase separation started (onset of phase separation). As soon as the phase separation was initiated by the on-going cure reaction, the point tracker stopped due to its termination criterion that only allowed a defined deviation of the initially targeted points. Here, diffusion in both directions was measured simultaneously with the point tracker algorithm. This became possible due to an increased contrast with respective to more robust feature detection for the diffusion of PEI into the reactive epoxy. Figure 6 shows four different time steps within a reactive multicomponent diffusion experiment. Images are included of the beginning and end of diffusion, the onset of phase separation and the equilibrium of phase separation. Through this representation of the interphase formation, it was then possible to quantify the accumulated interphase thickness depending on the stage of gradient interphase formation. A curing kinetic model (see Supporting Information) was used to compute the evolution of the degree of cure during the isothermal experiments.

**Figure 7:**
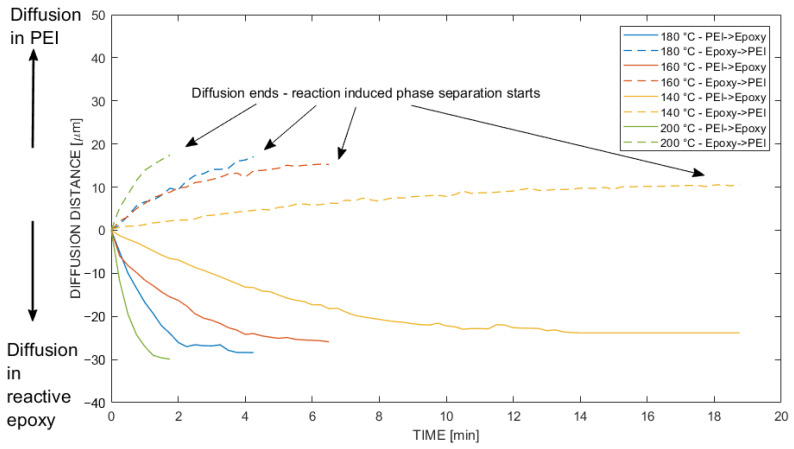
Diffusivity of reactive multicomponent diffusion experiments on an optical hot stage setup derived by the point tracker algorithm. Values in the positive direction indicate diffusion from the epoxy system into PEI, whereas values in the negative direction indicate diffusion from PEI into the epoxy system.

Figure 7 presents reactive multicomponent diffusion experiments in the temperature range from 140 °C to 200 °C, whereby the reaction-diffusion in the positive direction is indicated as ‘diffusion in PEI’ and vice versa. The diffusion speed depends on the directionality and temperature. Consequently, the interphase became larger as the temperature increased, since the diffusivity was higher. However, at some point, the interphase thickness reached a limit due to the advanced degree of cure of the thermoset. At this point, no further increase of the interphase thickness could be achieved. The relation of phase separation and cure temperature was characterized by Teuwen et al. [24], where at higher temperatures the phase separation would occur at a higher degree of cure. Figure 7 shows that PEI diffused into the epoxy at a much higher diffusion rate in comparison to the opposite direction.

The gradient interphase formation roughly consists of the following steps: First, dissolution, then diffusion and reaction followed by a reaction-induced phase separation. The mobility of both constituents (epoxy, PEI) is still high after triggering the phase separation. Therefore, phase separation enlarges the overall thickness of the interphase with decomposition of the morphology [9]. Table 2 summarizes the characteristics of interphase formation for reactive multicomponent diffusion at different temperatures at the final stage of diffusion and at the fully cured stage (see Figure 6). The lowest interphase thickness was found for the lowest cure temperature at 140 °C. When increasing the cure temperature to 160 °C, an increase of 7 μm was observed compared to 140 °C—from 160 °C to 180 °C around 5 μm, and from 180 °C to 200 °C around 2 μm. Consequently, the maximum achievable interphase thickness (after diffusion) of 47.29 μm was reached with a curing temperature around 200 °C. According to Teuwen et al. [24], the same conclusion was drawn with fully cured samples by measurements of the interphase thickness. This distinctively shows that PEI is an excellent thermoplastic material in combination with the used epoxy-amine system, as a maximum interphase thickness is reached within the designated curing cycles.

### 3.2. Mono and Reactive Multicomponent Diffusion Experiments with Raman Spectroscopy

**Figure 8:**
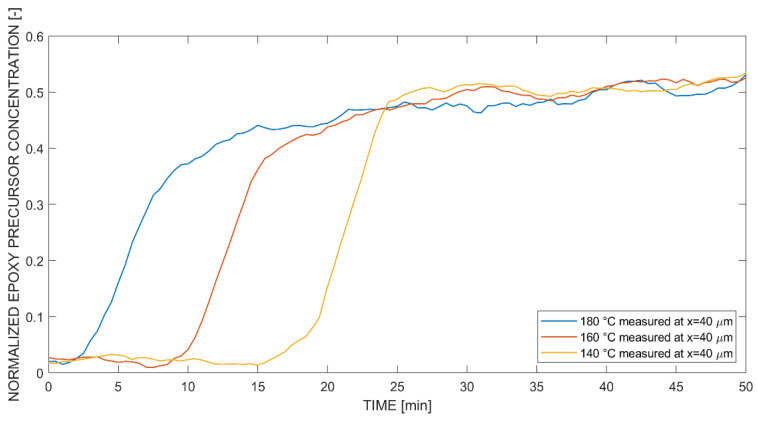
Temperature-dependent concentration curves normalized to Raman intensity amplitude 1819@988 cm^−1^ at coordinate x = 40 μm (positive) for diffusion of the (monocomponent) epoxy precursor into PEI.

Figure 8 shows the time-resolved and normalized epoxy precursor concentrations into PEI, measured with in situ Raman spectroscopy acquired at a distance of 40 μm (positive) from the original interface for isothermal temperatures of 140 °C, 160 °C and 180 °C. The diffusion front reached the x-location at different time steps due to increased diffusivity at higher temperatures. The fast concentration increases at the time where the epoxy precursor reaches the measurement position was attributed to a strong swelling mechanism. This is due to the high free volume of the PEI. After the concentration increase, all curves increased linearly to a similar concentration level at 50 min. The linear increase of the concentration was another dominant characteristic. Therefore, the stated observations were implied to behave according to non-Fickian (Case II) diffusion, which is typical in glassy polymers subjected to penetration by a low-molecular weight solvent [29]. Nonetheless, further information such as the diffusion kinetics is needed for verification of the stated transport phenomena. Despite this fact, the apparent phenomena are referred to as non-Fickian diffusion.

**Figure 9:**
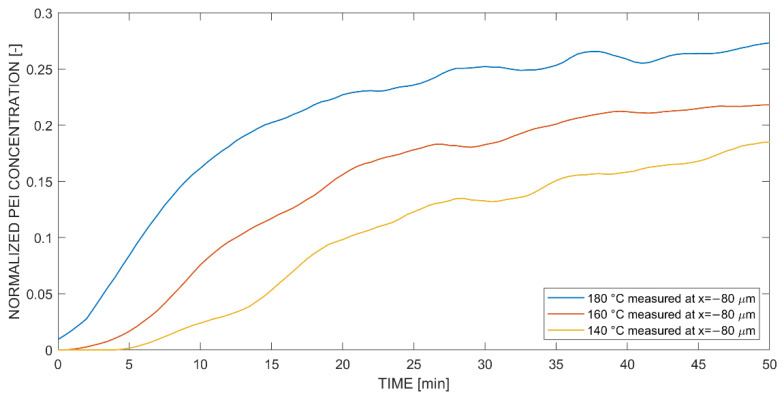
Temperature-dependent concentration curves normalized to Raman intensity amplitude 2437@1003 cm^−1^ at coordinate x = −80 μm (negative) for diffusion of the PEI into (monocomponent) epoxy precursor.

Figure 9 presents normalized concentration curves of PEI into epoxy precursor at a defined negative position of –80 μm for isothermal temperatures of 140 °C, 160 °C and 180 °C. The difference in diffusion behavior compared to Figure 8 is visible. Fickian diffusion was dominant within this area, as no swelling mechanism was apparent. This is further supported by analyzing the shape of the curve. First, the concentration does not show a pronounced increase but rather a distinct change in slope. Second, different temperatures led to different maximum concentration values at 50 min. This was a key characteristic that describes the Fickian diffusion because the concentration rate is proportional to the diffusion flux. Additionally, it is interesting that Fickian diffusion was not apparently visible within the optical hot stage experiments. On one hand, this is explained by the strongly pronounced and swelled diffusion front of non-Fickian diffusion in positive direction, whereby a significant change in concentration is visible. On the other hand, a more gradual change in concentration is apparent in Fickian diffusion, which concludes in a slighter change in color or refractive index in the negative direction of optical diffusion experiments with epoxy precursors (see Figure 4). This is supported by the overall lower concentrations of Fickian diffusion (Figure 9) in comparison to non-Fickian diffusion (Figure 8).

**Figure 10:**
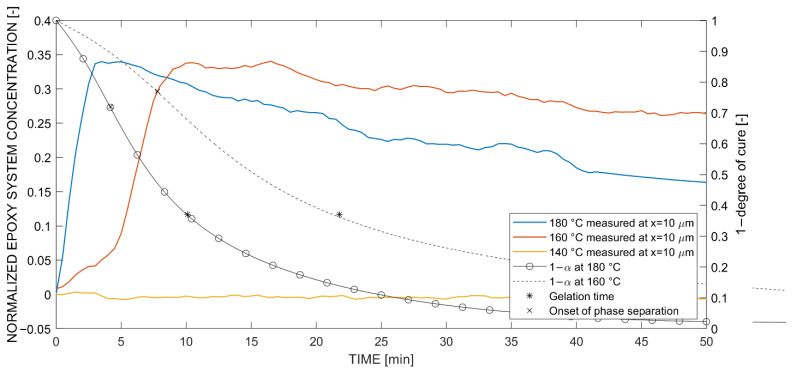
Temperature-dependent concentration curves normalized to Raman intensity amplitude 4504@988 cm^−1^ at coordinate x = 10 μm (positive) for diffusion of the (reactive multicomponent) epoxy system into PEI.

Next, reactive multicomponent diffusion experiments were performed with in situ Raman spectroscopy. For this, the full epoxy system (TGMAP-DGEBF-DDS) was homogenously mixed and used for the measurements. Again, measurements were performed in both positive and negative x-directions with respect to the thermoplastic edge. First, Figure 10 presents the normalized concentration curves in the positive direction, from the epoxy system into PEI. Additionally, the degree of cure α over time was overlaid as a 1−α function, whereby the gelation time was marked (*) to highlight the interaction between reaction and diffusion. This indicates the time when a significant increase of the molecular weight led to lower diffusion rates in the epoxy system. The gelation time as a function of temperature (Arrhenius dependency) was tested experimentally on a Kofler heating bench. The following expression was derived:

(1) $$t_{gel}=7.85\;10^{-9}\exp\left(77,974.3\;\left[J/mol\right]/R_{gas}\;T\right)\;\left[\min\right],$$

where $R_{gas}$ is the universal gas constant, T is the isothermal temperature and $t_{gel}$ is the gelation time in minutes. The results show a gelation time at degree of cure α=0.63 ± 0.05, which correlates with Hein [58] and Rajagopalan [40], who both investigated comparable high temperature epoxy-amine systems (HexFlow^®^ RTM 6, HexPly^®^ 3501-6).

Figure 10 shows the swelling mechanism, seen due to a sudden increase in concentration when the epoxy system reached the measurement location, which was also identified in monocomponent diffusion experiments into PEI. The measurement at 140 °C did not experience any change in concentration because the diffusion front did not reach the measuring position at x = 10 μm. This was also observed in Figure 7, where the reaction-diffusion terminated before 10 μm. Furthermore, both measurements at 160 °C and 180 °C behaved similarly, where after the swelling, a maximum concentration was reached and sustained, followed by a linear decrease to a final concentration at 50 min (180 °C: f(t) = −0.004t + 0.3467, 160 °C: f(t) = −0.0021x + 0.3579). An in-detail discussion concerning this phenomenon is provided in the discussion section.

**Figure 11:**
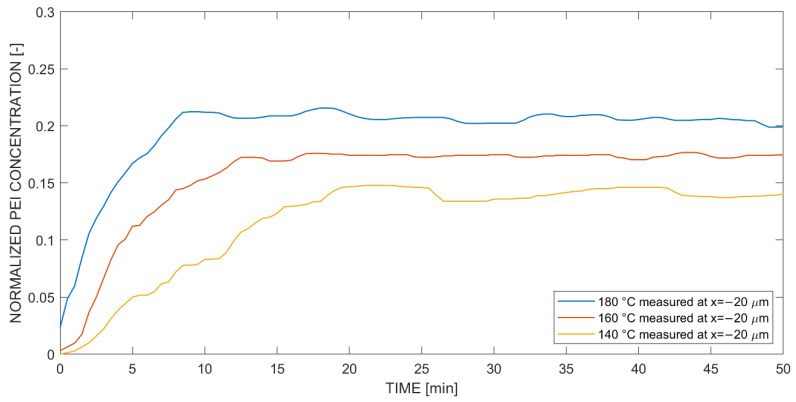
Temperature-dependent concentration curves normalized to Raman intensity amplitude 2437@1003 cm^−1^ at coordinate x = −20 μm (negative) for diffusion of the PEI into (reactive multicomponent) epoxy system.

Next, the in situ diffusion experiment in negative direction, from PEI into the epoxy system, was analyzed (see Figure 11). Again, Fickian diffusion was apparent due to the higher change in concentration slope at higher cure temperatures. As the epoxy system underwent curing, the increase in concentration terminated early, i.e., earlier than observed in Figure 9, and reached an equilibrium level, i.e., where no increase of concentration was observed. The diffusion in the opposite, negative direction was more distinctive, respectively faster than in the positive direction and was already seen with optical hot stage microscopy (see Figure 7). Nevertheless, the concentration range was smaller, which means that the developing concentration gradient was less steep in Fickian diffusion.

## 4. Discussion

### 4.1. Detailed Analysis of Reactive Multicomponent Diffusion from the Epoxy System into PEI

The linear decrease of the reactive multicomponent diffusion from the epoxy system into PEI (Figure 10) was attributed to two main sources. An assumption that applies well to the behavior in Figure 10 is volumetric changes of the epoxy system during curing. Volumetric changes of the epoxy-amine system that occur during curing are influenced by a combination of thermal and chemical effects. Here, the volumetric cure shrinkage of the thermoset resin took place during the formation of its chemical network and was the result of an increasing crosslink density. Khoun et al. [59] showed that shrinkage corresponds to linear relationships between the volumetric shrinkage and the degree-of-cure before and after the gelation. At the gelation transition, a change in the shrinkage rate was detected. Consequently, it could be a valid explanation that the volumetric concentrations of epoxy into PEI in Figure 10 decreased linearly and led to different end values at 50 min, which were 0.16 for 180 °C and 0.26 for 160 °C.

Another influence is the change in Raman scattering due to the on-going polymerization of the thermoset. Lyon et al. [60] and Hardis et al. [61] investigated the in situ cure monitoring of an epoxy resin with Raman spectroscopy and compared it with other characterization techniques. They attributed the epoxide ring to the Raman peak at 1252 cm^−1^, whereby its intensity decreased with time, corresponding to the opening of the epoxide ring during the epoxy-amine reactions. Other Raman peaks, e.g., 1112 cm^−1^ associated with resin backbone vibrations, remained constant during the reaction and could be used as a reference when observing the degree of cure [60,61].

**Figure 12:**
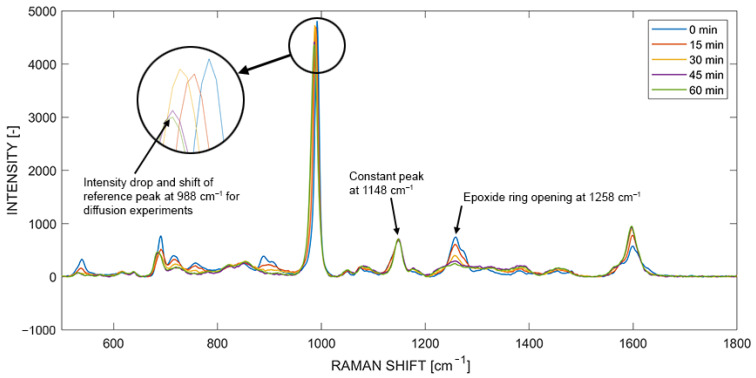
In situ Raman spectra featuring the reactive and reference peaks during curing at 180 °C.

Therefore, in situ Raman spectroscopy measurements were performed with pure mixed epoxy resin as a reference, whereby the same parameters as stated in Table 1 were used. Figure 12 presents time mapped results at 180 °C cure temperature. It can be clearly seen that the peaks at 1601 cm^−1^ and 1258 cm^−1^ in the experiment did not remain constant, whereas the peak at 1148 cm^−1^ remained constant. Furthermore, the reference peak (988.2 cm^−1^) for mono and reactive multicomponent diffusion experiments shows a significant drop in intensity during curing. This led to the conclusion that the apparent drop in concentration seen in reactive multicomponent diffusion seemed influenced by both volumetric shrinkage and the change in Raman scattering intensity due to the on-going curing reaction.

### 4.2. Comparison of Optical and Spectral Experimental Monocomponent Diffusion Results

A comparison of optical hot stage experiments and in situ Raman experiments of different measurement positions was made, as the actual starting points of the experiment for both methods were recorded and thus used as a reference. The comparison was of high interest because the optical diffusion front only appeared due to influences such as contrast and refractive index. As the point tracker algorithm measures relative motion of the diffusion front, it was not clear how the time- and temperature-dependent concentration gradient develops and changes over time and influences the resulting traced points. Hence, the comparison indicates how accurate the optical measurements were in relation to the spectral measurements. The in situ Raman experiments were considered more representative because relative changes in concentrations were measured. The measurement error of the starting point was approximately ±5 s, and only monocomponent diffusion experiments were compared, as reactive multicomponent diffusion experiments showed an increased complexity.

**Figure 13:**
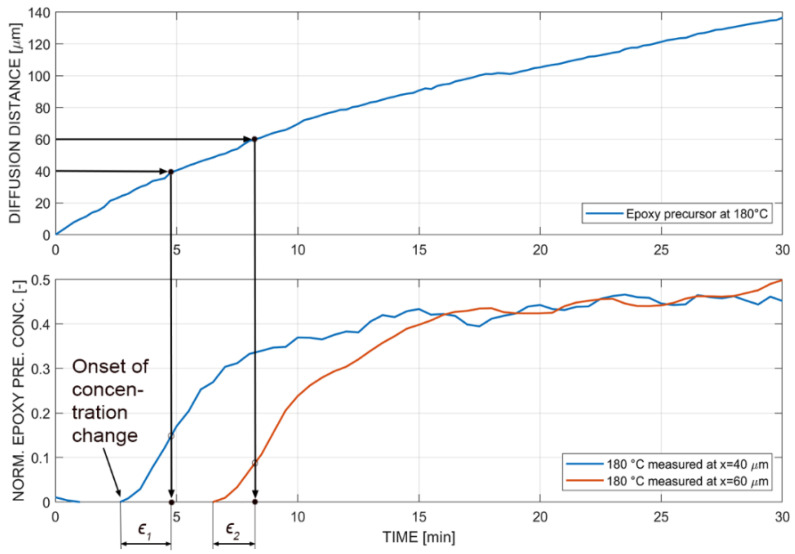
Comparison of in situ Raman diffusion measurements with optical hot stage experiments with the epoxy precursor.

Figure 13 presents an overview of the procedure performed to compare the two methodologies: First, the measured position in the in situ Raman experiments was read and assigned in the curve derived by the optical hot stage microscopy coupled with the point tracker. This resulted in a time step for optical measurements that can be compared to the spectral measurement, whereby the onset of concentration change indicates the corresponding time step. The time step of the onset of concentration change was defined if the condition of a 1% signal increase was fulfilled. The difference in time between optical and spectral measurements is further referred to as concentration onset error ($\epsilon_{1}$ for 40 μm and $\epsilon_{2}$ for 60 μm at 180 °C in Figure 13).

**Figure 14:**
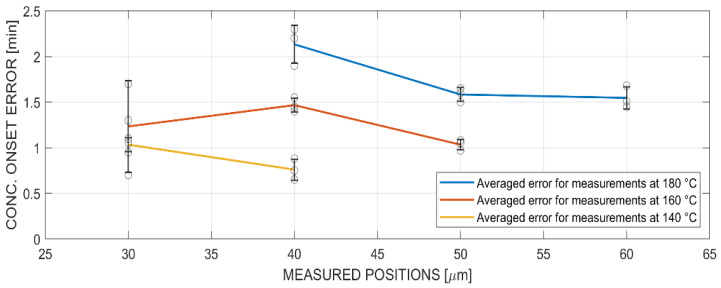
Comparison of the concentration onset error at 180 °C, 160 °C and 140 °C at different positions.

The concentration onset error was evaluated for all measured temperatures and positions and summarized in Figure 14. Each temperature and position are represented with a mean and standard deviation of three measurements. The optical measurements showed an incorrect onset of the concentration gradient, because the concentration curves were already at a higher concentration range than zero (see Figure 13) to create optical features for the point tracker detection. Additionally, it was possible to identify a tendency of the concentration onset error to be significantly higher at higher temperatures. It was interpreted that the time- and space-dependent concentration gradient had a different shape at different temperatures, and therefore the contrast and refractive index appeared differently in optical measurements. In this case, the 140 °C optical experiments were more accurate than the in situ Raman experiments. Furthermore, there was an influence of the measured position, because the error decreased at increased measurement positions. This was attributed to the shape of the concentration gradient that evolved and represented the diffusion front. The concentration value shifted to lower values at higher position values, which indicated that the concentration gradient was stretched or elongated in the x-direction with higher position values. Despite the performed comparison, an in-detail investigation is needed that evaluates further existing influences, such as the limit of detection and limit of identification [55].

## 5. Conclusions

This study presents methods to characterize in situ diffusion and reaction-diffusion mechanisms during the formation of gradient interphases between PEI and the epoxy system (TGMAP-DGEBF-DDS). Experiments were performed with a non-reactive monocomponent (only epoxy precursor with PEI) and reactive multicomponent (epoxy system with PEI) setup. Two methods were introduced, namely optical hot stage microscopy coupled with a point tracker algorithm, and in situ Raman spectroscopy. It was possible to identify Fickian diffusion from PEI into the epoxy precursor with respective to the epoxy system and non-Fickian diffusion in the vice versa case. Fickian diffusion is present due to the entanglement of polymer chains. Non-Fickian diffusion is present due to extensive swelling and an increase in volume during dissolution. Despite this fact, the non-Fickian mechanism needs additional investigation to prove the type of diffusion. Both mechanisms were dominant throughout all measured positions and temperatures. Furthermore, both developed methodologies enabled a qualitative description of the diffusion rate by means of the magnitude of concentration of its constituents, e.g., the diffusion front propagated faster but with lower concentration of PEI for the Fickian diffusion experiment. It was feasible to characterize both monocomponent and reactive multicomponent system, whereby the latter presented an increased complexity (influence of the reaction and chemical shrinkage) that needs further investigation.

A detailed discussion related to the comparison of optical and spectral measurements showed differences in in situ characterization due to the change in the optical appearance of the evolving and propagating concentration gradients. Compared to the optical measurements, time-resolved in situ Raman experiments presented more reliable and trustworthy results, as relative changes in the spectral response were measured in real-time.

This study allowed an in-depth investigation of the initial phase of interphase formation of PEI with an epoxy system. Investigating the diffusivity of monocomponents and their dependency on time and temperature and the diffusivity of reactive multicomponent systems contributed to a better understanding of the underlying mechanisms. Future work will focus on characterizing the reactive multicomponent system, including phase separation with in situ Raman experiments.

## Figures and Tables

**Table 1:** Experimentally-determined parameters for Raman spectroscopy for monocomponent and reactive multicomponent in situ diffusion experiments.

Parameter	Value	Parameter	Value
Laser	785 nm	Hole	500 μm
Filter	50%	Slit	100 μm
Lens	x100	Range	600–2000 cm^−1^
Accumulations	2	Acquisition time	2 s
Grafting	1200 (750 nm)	Delay time	0 s
Total time	3000 s	Time interval	30 s
Autofocus mode	Off	Repetitive mode	Off

**Table 2:** Overview of important values derived from reactive multicomponent diffusion experiments.

Temperature (°C)	Maximum Diffusion Length of PEI (μm)	Maximum Diffusion Length of Epoxy (μm)	Interphase Thickness after Diffusion (μm)	Time at Max. Diffusion (min)	Measured Interphase of Cured Samples (μm) [9,24]
200	29.89	17.39	47.29	1.95	82.3
180	28.40	16.95	45.36	4	80.7
160	25.89	15.31	41.21	7.5	62.2
140	23.81	10.71	34.52	18	50.7

## Data Availability

The data presented in this study are available upon request from the corresponding author.

## Supplementary Materials

The following supporting information can be downloaded at https://www.mdpi.com/article/10.3390/polym14030435/s1, Equations (S1)–(S7): Governing equations, Figures S1 and S2: Comparison of experimental data and fitted model, Table S1: Cure kinetics model parameter.
