# Supplementary material for: In Situ Characterization of the Reaction-Diffusion Behavior during the Gradient Interphase Formation of Polyetherimide with a High-Temperature Epoxy System
Source: Polymers (Basel). 2022 Jan 21;14(3):435. doi: 10.3390/polym14030435 (PMC8839078; doi:10.3390/polym14030435)
Supplement: Supplementary file 1 [file polymers-14-00435-s001.zip › polymers-1563199-supplementary.pdf]

Supplemental Material

Development of the curing kinetic model

The cure kinetics is derived by isothermal and dynamical differential scanning calorimetry (DSC) measurements. Here, the reaction enthalpy is calculated by integrating the heat flow for a specific time frame with a satisfactory baseline. The degree of cure is linked to the reaction enthalpy according to

$$\alpha(t) = \frac{H_r(t)}{H_{r,tot}}, \tag{S1}$$

whereby $\alpha(t)$ is the degree of cure and $H_r(t)$ the reaction enthalpy at the moment t and $H_{r,tot}$ is the total reaction enthalpy. Its derivative with respect to time characterizes the rate of cure – here, many approaches have been introduced such as standard Kamal-Sourour [1], Kamal [2] and Karkanas [3]. At a certain point the epoxy resin starts to crosslink, resulting in the release of an exothermic heat flow. The complete infolding area of the exothermic peak is defined as the total reaction enthalpy $H_{r,tot}$.

There are many suggestions in literature for modelling the cure behavior. The simplest approach is a Kamal-Sourour model which describes the autocatalytic reaction with

$$\frac{d\alpha}{dt} = k\alpha^m(1-\alpha)^n \, , \tag{S2}$$

whereby m and n are parameters of adaption and k is the Arrhenius constant

$$k = A \exp\left(\frac{-E_a}{R_{gas}\,T}\right), \tag{S3}$$

whereby A is a scale factor, E_a is the activation energy, R_{gas} the universal temperature and T the actual temperature. The Kamal-Sourour, Kamal and Karkanas models do not include a diffusion controlled reaction [4]. This means that chemical controlled reaction is modelled well, but after reaching the vitrification point the curing is mainly influenced by diffusion. Therefore, models have been introduced with an empirical diffusion term, in order to satisfy both chemical and diffusion-controlled curing. Nevertheless, this study introduces an alternative model. It is based on a temperature dependent maximum degree of cure. Here, the value of the degree of cure is between 0 and α_{max} , instead of 0 and 1. The Karkanas model [3] is used with the approach

$$\frac{d\alpha}{dt} = k_1(\alpha_{max} - \alpha)^l + k_2\alpha^m(\alpha_{max} - \alpha)^n \quad \text{(S4)}$$

with

$$k_i = A_i \exp\left(\frac{-E_{Ai}}{R_{gas}T}\right) \; with \; i = 1{,}2 \quad \text{(S5)}$$

Furthermore, the approach of the temperature dependent maximum degree of cure is nonlinear [5]. In more detail, the maximum degree of cure is s-shaped in respect to the cure temperature and is defined according to

$$\alpha_{max} = \frac{1}{1 + \exp\left(-T\, a_{max} + b_{max}\right)} \; , \quad \text{(S61)}$$

whereby a_{max} and b_{max} are parameters which fit to the experimentally generated data by DSC. In fact, isothermal DSC experiments to identify the maximum degree of cure. The sample is cured isothermally to the point of no change in the exothermic reaction rate. Afterwards, the sample is rapidly cooled to room temperature followed by a dynamic heat ramp to measure the rest enthalpy. Based on the rest enthalpy and the total reaction enthalpy, it is possible to calculate the temperature dependent maximum degree of cure

$$\alpha_{max}(t,T) = 1 - \frac{H_{r,rest}(t,T)}{H_{r,tot}} . \quad \text{(S7)}$$

Under the assumption that the current reaction enthalpy behaves proportional to the degree of cure, the DSC measurements were used with a non-linear optimisation strategy in Matlab to optimise the data to the modified Karkanas model according to expression (S4). Hence, the objective function is defined as a minimisation problem to find the smallest difference between experimental data and the model.

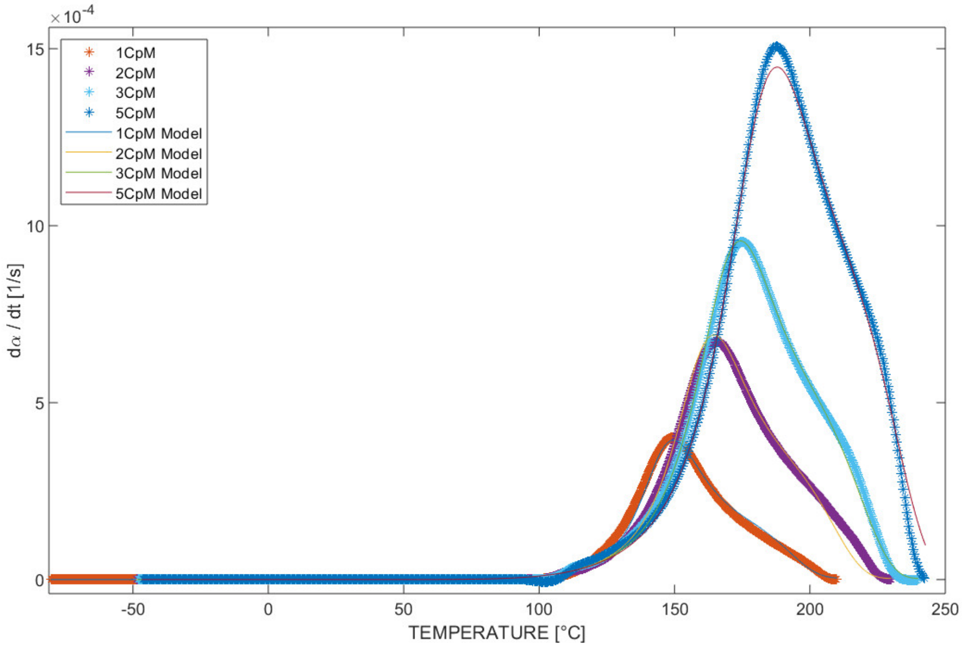

Figure S1 Comparison of experimental data and fitted model for different heating rates

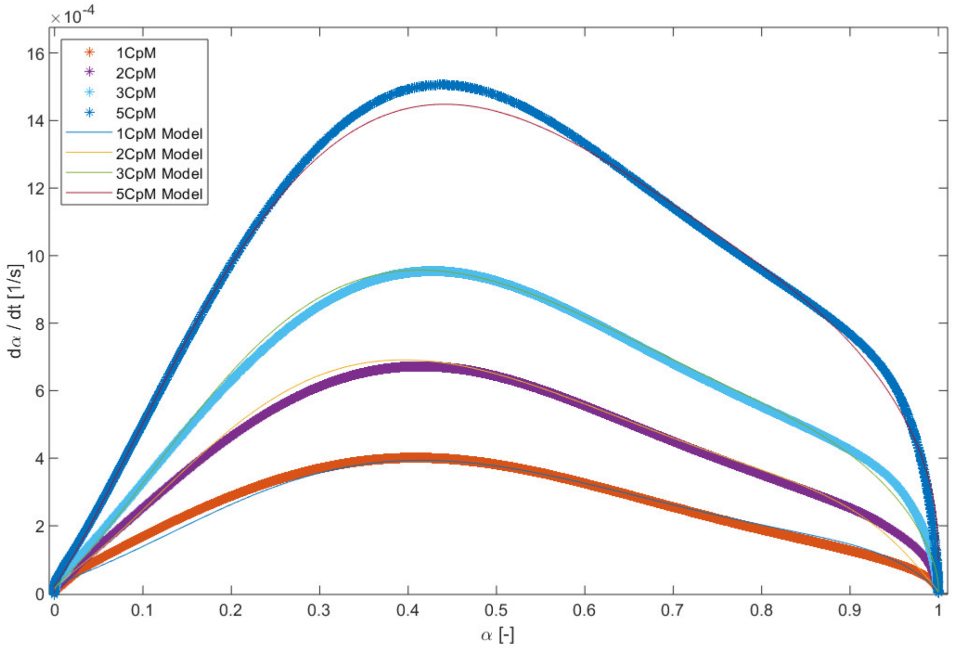

Figure S2 Comparison of experimental data and fitted model for different heating rates

The fitted model shows a suitable representation of the experimental data (Figure S1 and Figure S2) and was used as a basis for the reaction-diffusion investigation. Table S1 summarizes the developed Karkanas model with the fitted parameters derived from the used optimization technique.

Table S1 Results of optimized parameters settings for cure kinetics model

Parameter	Value
A_1 [s^{-1}]	167788.09
E_1 [kJ mol^{-1}]	71724.24
A_2 [s^{-1}]	64.48
E_2 [kJ mol^{-1}]	31612.11
m [-]	1.561
l [-]	0.821
n [-]	3.261

References

1. Kamal, M.R.; Sourour, S. Kinetics and thermal characterization of thermoset cure. *Polym. Eng. Sci.* **1973**, *13(1)*, 59-64. doi:10.1002/pen.760130110
2. Kamal, M.R. Thermoset characterization for moldability analysis. *Polym. Eng. Sci.* **1974**, *14(3)*, 231-239. doi:10.1002/pen.760140312
3. Karkanas, P.I. Modelling the cure of a commercial epoxy resin for applications in resin transfer moulding. *Polym. Int.* **1996**, *41(2)*, 183-191.
4. Garschke, C.; Parlevliet, P.P.; Weimer, C.; Fox, B.L. Cure kinetics and viscosity modelling of a high-performance epoxy resin film. *Polym. Test.* **2013**, *32(1)*, 150-157. doi:10.1016/j.polymertesting.2012.09.011
5. Hein, R. Vorhersage und In-Situ Bewertung fertigungsbedingter Deformationen und Eigenspannungen von Kompositen. Dissertation, Deutsches Zentrum für Luft- und Raumfahrt e.V.: Braunschweig, Germany, 2019.
